# 

**Published:** 1810-05

**Authors:** 


### To CORRESPONDENTS.

We would very readily have afforded a place to any recital of facts *an impartial Practitioner* might have sent us, on the subject of Vaccination, whichever side of the question they might have tended to support; but we cannot spare room for long declamatory arguments If this Practitioner really doubts the occasional occurrence of Small-pox a second time, and gives such easy credence to erroneous statements against vaccination, as he appears by his Letter to do, we fear his bias to one side of the question is too great to entitle him to the character of *Impartiality.*

